# Assessment of the Effect of Solid Waste Dump Site on Surrounding Soil and River Water Quality in Tepi Town, Southwest Ethiopia

**DOI:** 10.1155/2020/5157046

**Published:** 2020-06-08

**Authors:** Besufekad Mekonnen, Alemayehu Haddis, Wuhib Zeine

**Affiliations:** ^1^Department of Public Health, Mizan-Tepi University, Mizan-Aman, Ethiopia; ^2^Department of Environmental Health Science and Technology, Jimma University, Jimma, Ethiopia

## Abstract

An increase in urban population and the rising demand for food and other essentials perpetuate a rise in the amount of waste being generated daily by each household. In Ethiopia, this waste is eventually thrown into open dump sites. It can cause severe impact on soil and surface water quality. As a result, it becomes the probable source of human health risk through food chain. Therefore, this study was aimed at assessing the effect of a solid waste dump site on surrounding soil and river water quality in Tepi town, Southwest Ethiopia. Three surface water, one leachate, and four soil samples were collected and analyzed. Six heavy metals for surface water and leachate samples and four heavy metals for soil samples were measured by flame atomic absorption spectroscopy. In addition, physiochemical parameters were analyzed using standard methods. The data were analyzed statistically using Origin pro version 8.0 computer software packages. The pH of soil was slightly basic ranging from 8 ± 0.1 to 8.7 ± 0.21. Electrical conductivity was lower at 60 meters (1800 ± 0.5 *μ*s/cm) and higher in the other sample sites (3490 ± 0.66–4920 ± 1.04 *μ*s/cm). The concentration of heavy metals in soil samples revealed cadmium (0.53 ± 0.01–2.26 ± 0.02 mg/kg), zinc (623.93 ± 0.29–859.41 ± 0.02 mg/kg), lead (3.26 ± 0.25–57.560.26 mg/kg), and copper (204.06 ± 0.06–337.11 ± 0.01 mg/kg). Lead, cadmium, manganese, nickel, copper, and zinc were found in the leachate water; nickel and manganese were found in the nearby river water; BOD_5_ and COD for both leachate and stream water samples were found to be higher than standard guideline values. The finding suggested that solid waste open dump site adversely affects soil and water quality in the study area and becomes a probable source of risk for human health via the food chain.

## 1. Introduction

Thousands of tons of solid waste are generated daily in African countries [1, 2]. In Ethiopia, less than half of the solid waste produced is collected and 95 percent of that amount is either indiscriminately thrown away at various dumping sites on the periphery of urban centers or at a number of so-called temporary sites and typically empty lots scattered throughout the city [3]. The indiscriminate and open disposal of waste can cause environmental degradation through introducing different toxicants including heavy metals in the soil and water compartments [4, 5].

Surface water contamination plays a significant role as a population stressor because; human beings are dependent on water for their existence. Rainfall events may alternately dilute toxicity or increase it if the rate of transport increases the flow of contaminants to surface water. Rivers and streams are sinks for municipal solid wastes. Wastes are most often discharged into the receiving water bodies with little or no consideration to their assimilative capacities [6].

Open dumping of municipal solid waste is a common practice in Ethiopia, and the problem of solid waste disposal is one of the major problems of the community and municipalities [7, 8]. A recent study shows that in most towns, municipal solid wastes are disposed of in open spaces without discriminating major residential areas, roadsides, drainage areas, rivers, riversides, and forests. This leads to the introduction of hazardous substances including heavy metals into water and soil ecology [4, 9].

Nevertheless, there is a need for a comprehensive and detailed study about the content of heavy metals and the physiochemical properties of soil and surface water around solid waste disposal facilities in Ethiopia. There are suggestions for further studies on heavy metals content in the soil profile and surface water closer to dump sites [5, 9, 10]. The presence in excess of heavy metals in soil and water adversely affects soil microbes, ground, and surface water quality and ultimately becomes harmful to the health of humans in the food chain [11, 12].

Similarly, Tepi town is characterized by rapid population growth caused by natural increase and migration. Such a rapid increase in population together with the rapid development of the town has produced increasing volume of solid waste. Indiscriminate solid waste disposal is actually a menace and embarrassment to Tepi town.

Furthermore, solid waste generated in Tepi town is disposed at an unapproved dump site having a size of 10,000 m^3^ and with an average height of 2.5 meters. Waterways (natural surface drainage line) which drain to the nearby agricultural field potentially pose adverse public health and environmental impact. Moreover, the dump site is surrounded by stream. The area around the dumpsite is free of informal settlers at the moment, and there is no grazing land either. The dump site is operational and serves as the main landfill site for Tepi town municipality. Although no settlers in the immediate vicinity, it has been an established fact that solid waste poses various threats to public health and adversely affects soil and water quality especially when it is not appropriately disposed of [13].

Due to high rainfall experienced in the study area (mean annual rainfall is between 1700–2200 mm), the dump site becomes washed out and the leachate with its pollutants drains into the Shay Wenz River. Fresh water is an imperative resource for people and requires so many provisions such as regulatory and cultural framework and enhancement of ecosystem services for the community and the world in general [14]. Similarly, the river near to Tepi town solid waste dump site is largely used by the local community for irrigation, bathing, and drinking purpose.

To date, there is no study conducted to show the extent of pollution status in the soil, surface water, and leachate adjacent to solid waste dump sites and compared with national and international guideline values. Therefore, this paper is aimed at assessing the effect of open dumping of solid waste on surrounding soil and river water quality in Tepi town, Southwest Ethiopia.

## 2. Materials and Methods

### 2.1. Study Design

Tepi is a town in Southwest Ethiopia and well known by the production of coffee and spices. The town is located at 621 km south of Addis Ababa with latitude and longitude of 7°12′N 35°27′E, respectively, and with a mean elevation of 1,097 meters above sea level. According to Ethiopian Central Statistic Authority population projection of 2017, the total population of the town was 60,160 [15]. An experimental study was used to characterize the leachate quality and to determine the quality of soil and surface water in the nearby dumpsite. The study was conducted from March 1 to June 30, 2017.

### 2.2. Soil Sample Collection and Treatment

**Figure 1:**
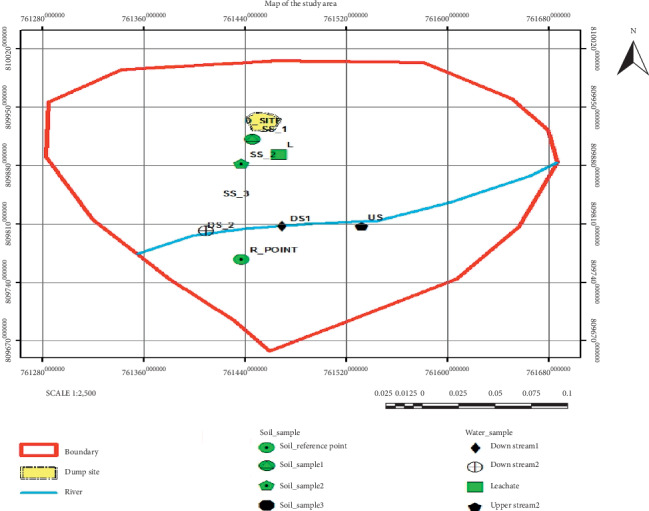
Sampling points at Tepi town solid waste dumpsite, Southwest Ethiopia.

The sample sites were selected by transects through simple random sampling method towards gully erosion based on the United States Environmental Protection Agency soil sampling protocol [16, 17]. The sample points were located at 10 meters, 30 meters, and 60 meters away from the periphery of dump site as indicated in Figure 1 [5, 16, 18, 19]. Soil samples were collected from the dump site by stainless steel hand augur [18, 20]. The samples were taken at a depth of 0.5–20 cm from each sample point. The top 0.5 cm of surface soil was removed before the samples were taken [5, 18, 20]. Meanwhile, the representative samples were coded and labeled on the information sheet and attached to the sample polyethylene bag. Then, the collected samples were thoroughly mixed on the net polyethylene sheet and transported to the laboratory which were then air dried for 72 hours [5, 18].

The soil samples were disaggregated with mortar and pestle, finely powdered, and thoroughly mixed together with other precautions to prevent contamination of the samples. Then, air-dried soil sample were grinded crushed and sieved through a 2 mm sieve, and <2 mm mesh size was used for analysis. The soil pH and electrical conductivity, heavy metals, and organic matter were detected [5, 18, 21].

### 2.3. Water Sample Collection and Treatment

Water samples were taken by the purposive sampling technique. Optimum amount of river water samples (1-liter) were collected from three different sampling points, namely, upper stream (US) 100 meters far from the dump site, near to the dump site (DS1) in reference to the leachate outlet, and 100 meters far from the dump site in downstream direction as shown in Figure 1 (DS2) [18, 22, 23].

These water samples were taken from the places where the river has laminar flow pattern in order to keep uniformity of samples and obtained at a depth of 10–15 cm below the surface water to avoid floating debris and filled into 1-liter polyethylene bottles. The leachate sample (L) was taken from the place near the dump site as indicated in Figure 1 [18, 22, 23]. At each sampling point, two sets of water samples were collected, and 2.0 ml of concentrated HNO_3_ was added to one of the bottles to bring the pH < 2 in order to prevent adsorptions of heavy metals on the bottom of sample containers. The acidified samples were used for elemental analysis and the nonacidified samples were used for biological analysis [18, 24, 25].

### 2.4. Analytical Methods

#### 2.4.1. Soil Sample Analysis

Soil electrical conductivity was analyzed by 1 : 2.5 soil-to-water extraction methods [20]. The extract was measured by a digital EC meter (H12300 EC/TDS/NaCl meter, HAWA instrument, Romania), and pH was measured with the pH meter (pH-016 model) by using a glass electrode. Determination of organic carbon in the soil was carried out through the spectrometric method of modified ISO 14235 [26]. Percent of organic carbon was converted to percent of organic matter, by using a conversion factor of 1.724 [27]. Heavy metals (lead, cadmium, copper, and zinc) extraction from soil samples was performed by an aquaregia digestion based on ISO 11466-recommended methods [17, 27], and the concentration of elements was measured by flame atomic absorption spectroscopy (PG 990, China model).

#### 2.4.2. Water Sample Analysis

In situ measurement of different parameters was done by using a digital portable multiparameter probe (Micro 800 plain test, UK model). In addition, electrical conductivity (EC) and total dissolved solids (TDSs) were measured by using the EC/TDS meter (Micro 800, Plain test, Wage tech company, UK), and turbidity was measured by using the turbidity meter (plain test, UK model).

At laboratory level, nitrate, sulfate, and fluoride were measured by the spectrometric method [24, 28–30], and the concentration was estimated by using the UV-visible spectrophotometer (Plain test 7500, Wag Tech Company, UK model).

The amounts of copper, zinc, lead, cadmium, nickel, and manganese were analyzed through the digestion procedure of the APHA 3111c air/acetylene oxidizing flame method [31], and the concentration of total elements was measured by flame atomic absorption spectroscopy (PG-990, China model). The dissolved oxygen content in the sample was measured by using azide modification of the titrimetric-iodometric method (Section 4500-O.C), and COD was determined by potassium dichromate in an open reflux method [32–35].

### 2.5. Data Analysis

The data were analyzed statistically using Origin pro version 8.0 computer software packages. Analysis of variance (ANOVA) was used to assess the significance difference between the mean values of heavy metals and physicochemical parameters in soil and water samples. Possibilities less than 0.05 (*p* < 0.05) were considered statistically significant. The analyzed data were presented by using tables. The mean values were compared with the limits in soil and water prescribed by the Ethiopian Environmental Protection Agency, United State Environmental Protection Agency, and WHO standards.

## 3. Results

The physiochemical properties of leachate sample and river water samples and heavy metal result of river water samples, leachate sample, and soil samples at Tepi town solid waste dump site along different sample locations and guideline values are provided in Tables 1–5.

## 4. Discussion

### 4.1. Physiochemical and Biological Parameters of Leachate and Stream Water Samples

The study revealed an increase in water temperature along the course of leachate, downstream, and the point near to dump sites as indicated in Tables 1 and 2. This might be due to differences in altitude and the presence of the effluent released from the open dump site.

Higher pH (8.5 ± 0.11) was recorded from the leachate sample as shown in Table 1. This shows that the leachate was alkaline, and this was typical of the sample from aged wastes [25, 30, 34, 35]. Lower pH was recorded near to dump site and downstream sample sites (8.1 ± 0.11 and 8 ± 0.1), respectively. The higher range of pH indicates higher productivity of water. Other studies conducted in the solid waste dump sites of Nigeria, South Sudan, Sri Lanka, and Ethiopia substantiate this finding which shows slightly basic pH in the nearby stream [9, 35–37]. However, the mean values of pH in water samples varied between 7.6 ± 0.21 and 8.5 ± 0.1. The limit value prescribed by WHO was between 6.5 and 8.5, and the limit value prescribed by Ethiopia EPA was between 6 and 9.

The sample points of leachate and near to dump sites showed higher TDS values than the limit prescribed by the WHO standard (500 mg/l). On the other hand, the sample point of the upper stream recorded lower TDS values. This might be due to the effect of the dump site. The lowest mean value of turbidity was observed in the upper stream sample site (61.6 ± 0.01 NTU) as presented in Table 2 although it was above the limit prescribed by WHO standard value (25 NTU). It might be due to indiscriminate disposal of waste into the water bodies.

The higher turbidity in the other sites might be due to the influence of open dump site. The highest turbidity values were observed than those investigated in Jordan dump sites that revealed values between 13.4 and 4.7 NTU and between 40 and 160 NTU, respectively, in the nearby stream and leachate water [38, 39].

A high EC value was observed in leachate sample (391.35 *μ*S/cm) as presented in Table 1 which is indicative of the presence of high amount of dissolved inorganic substances in ionized form in and around solid waste dump site [40]. In addition, the higher value of EC is a good indicator of the presence of contaminants such as potassium and sulfate [11, 41]. When considering the average value of conductivity in the leachate sample, it was concluded that leachate had the high amount of ionizable material.

The result of conductivity in this study was lower than the other studies conducted in Addis Ababa, Ethiopia and Sri Lanka solid waste dump sites that show 1102 *μ*S/cm up to 3720 *μ*S/cm and 1136 *μ*S/cm respectively in the nearby stream [3, 37]. On the other hand, the result of this study was higher than a similar study conducted in Juba (South Sudan) where the average values of electrical conductivity indicated between 89 *μ*S/cm and 229 *μ*S/cm in the nearby stream [36].

The COD values were higher than the permissible limit in all samples as shown in Table 2. It indicates the stream water was highly polluted with the chemicals which might have resulted from the solid waste dump site and indiscriminate disposal of solid waste. Nitrate values in all the sites registered higher values than the natural background level of 0.23 mg/l. The presence of nitrate may be the result of waste being disposed of at the dump sites and indiscriminate disposal of solid waste into the water bodies. Thus, contamination of the water bodies with chemicals from the dump sites is likely to occur. It could be attributed to runoff from farms along the banks of the river which may contain organic fertilizers.

The values of nitrate in the study area were lower than those in the similar studies conducted in the solid waste dump sites of Ethiopia and Ghana showing concentrations between 2.0–2.2 mg/l and 4.18–30.8 mg/l, respectively, in the nearby stream [3, 11]. However, our finding was found to be higher from the studies in Accra, Ghana, which revealed nitrate concentration of 0.046 mg/l in the upper stream up to 0.418 mg/l in the downstream [30]. However, the findings revealed that all the sample sites did not exceed nitrate limit values prescribed by WHO and Ethiopian EPA of 20 mg/l and 50 mg/l, respectively.

Fluoride levels in all sites were within the limits prescribed by Ethiopian EPA (1 mg/l) and WHO (2004) guideline value of 1.5 mg/l except for the leachate (L) sample site with a mean value of 1.71 ± 0.01 mg/l. However, the remaining sites, especially the point near to dump site (DS1) and the downstream sample location (DS2), registered the highest concentration compared with the upper stream (0.8 ± 0.01 mg/l). This might be due to fluoride-containing materials such as wood preservatives, glasses, and enamel dumped to the open dump site and in the nearby stream in Tepi.

All mean values of sulfate were below the limits prescribed by Ethiopian EPA and WHO standards (200 mg/l). The values were lower than other findings in Akot city in India and Addis Ababa solid waste dump sites which varied between 263 and 62.8 mg/l and 53 and 342 mg/l, respectively [3, 39] in the nearby stream; but the values were higher than those of another finding in Accra (Ghana) dump site showing sulfate concentration between 0.2 mg/l in upper stream and 25 mg/l in leachate water [30].

The potassium concentration in our study in water samples was lower than S. Sudan (73 mg/l) but higher than the finding around Akot City in India which ranged between 15 mg/l and 5.1 mg/l [38]. BOD_5_ values in the study area were higher than the limits prescribed by Ethiopian EPA and WHO standards of 5 mg/l. In addition, they were higher than those in a similar study conducted in Accra, Ghana, which revealed a concentration of 1.25 mg/l up to 100 mg/l in the nearby stream and leachate samples, respectively [30].

### 4.2. Heavy Metals in Stream Water and Leachate Samples

As indicated in Table 3, the concentration of cadmium in all water samples was below the detection limit (<0.02 mg/l) and below the limit prescribed by Ethiopian EPA and WHO standards 0.005 and 0.003 mg/l, respectively, except for leachate sample (0.3 ± 0.01 mg/l). Other studies in Accra (Ghana) and India are consistent with the findings of this study showing the cadmium concentration in surface water near to the dump site to be less than 0.003 mg/l [21, 24, 30].

The concentration of copper in the study sites fall within this range except for the leachate sample as presented in Table 4. The highest value of copper was recorded above the limit prescribed by Ethiopian EPA and WHO standards (0.1 mg/l). It was also with a higher value than that in a similar study conducted in Accra (Ghana) dump site which revealed below 0.059 mg/l but lower than another finding in Sri Lanka where the values of copper in surface and leachate water near to dump site were found to be between 0.08 and 9.9 mg/l [30, 37].

The values of zinc in the study area were higher than those of another study conducted in Accra (Ghana) where the concentration of zinc nearby stream was below detection limit [24], however, lower than another finding in Sri Lanka which was reported to range between 0.1 and 9.9 mg/l in leachate water [37]. It might be due to discharges of smelter slag wastes and the use of commercial products such as fertilizers and wood preservatives that contain zinc disposed of in the water body and in the nearby dump site.

According to the Ethiopian environmental protection agency, the prescribed limit of zinc in surface water lies between 0.003 mg/l and 0.5 mg/l. Thus, the values of zinc in the study area revealed values between the limit prescribed by EEPA except for the leachate sample that exceeded the standard.

According to WHO, the standard value of a nickel is 0.02 mg/l. In this study, nickel values at the site near to dump site (0.08 mg/l) and the downstream locations (0.06 mg/l) were slightly greater than the permissible standard limit of WHO except for the upper stream, which exhibits below detection limits (<0.04 mg/l). These values were also lower than the limit prescribed by Ethiopian environmental protection agency (0.1 mg/l) except for the leachate sample. Higher concentrations might be due to indiscriminate disposal of nickel-containing solid wastes such as electroplating, zinc base casting, and storage battery in open dump site near to the river. A consistent study to our finding is reported in Sri Lanka with a nickel value ranging between 0.03 and 9.9 mg/l in the nearby stream and leachate water [37].

The leachate water had the highest lead concentration as shown in Table 4 compared with other streams. In addition, it contained high lead value than the permissible limits of the Ethiopian EPA and WHO standard (0.05 mg/l). It might be due to the quantity and constituents of municipal solid waste that contains lead contents such as electronic waste, lead batteries, lead-based paints, pipes, and plastics that are indiscriminately disposed of in the dump site. Other studies in India and Ghana provided consistent findings to this study in that lead values were between below detection limit and 0.07 mg/l, respectively, in the nearby surface water [21, 24].

The values of manganese at leachate and downstream sample sites were higher than the guideline values of Ethiopian EPA (0.3 mg/l) and WHO (0.1 mg/l). The presence of manganese might be due to indiscriminate disposal of solid waste and entrance of leachate to the river. In addition, a high amount of manganese may be due to waste containing dry cell batteries, paints, glasses, and ceramics that were disposed of in the open dump site and pollution from manganese dioxide cells for which the town has no controlled methods of disposal. Leachate and downstream sites registered the amount above prescribed limits of Ethiopian EPA and WHO standards. However, the finding was lower than another finding in Sri Lanka that revealed 2.7 mg/l in leachate water near to dump site [37].

### 4.3. Soil pH and Electrical Conductivity (EC)

Soil pH in at all sample sites was slightly basic as shown in Table 5. Similar studies conducted in Addis Ababa (Ethiopia), Accra (Ghana), Lagos (Nigeria), Maradi city (Niger Republic), and Adama (Ethiopia) revealed slightly basic pH (between 8.17 and 7.37) in the nearby solid waste dump sites. Such pH conditions might have happened due to soil with the high metallic burden [4, 5, 30].

The EC values of soil at Tepi town solid waste dump site indicate a significant presence of trace metal ions or ionizable materials in the dumpsite [33]. However, the mean values of EC found in this study were less compared with another similar study in Addis Ababa (Ethiopia) dump site. The difference may be attributed to variations in the composition of waste. This can be proved from findings on waste composition done in Addis Ababa and Dilla cities which reveal 41% to be demolishing wastes [5, 42], while Jimma city which has a relatively similar setup to Tepi town has reported low demolishing wastes (11%) compared with other waste streams [43].

### 4.4. Heavy Metals in Soil Samples

The heavy metal content in soil samples is presented on Table 5. The highest mean values of lead (57.56 ± 0.26 and 52.12 ± 0.02 mg/kg, respectively) were observed in 10 meters and 30 meters far away from the dump site, respectively. These values were higher than the limit prescribed by Ethiopian EPA standard (40 mg/kg) [44]. The movement of lead along the distance was slightly towards the nearest of the periphery of the dump site. It may be due to differences in soil pH and organic matter.

The values of lead in this study area was lower compared with other studies done in Addis Ababa, India, and Maradi city (Niger Republic) dump sites which shows 17–852 mg/kg, 42.9–1833.5 mg/kg, and 79.133 mg/kg, respectively [4, 34]. It was higher than another study conducted in Adama city (Ethiopia) dump site which shows 1.033 mg/kg [5]. The difference may be due to variations in the quantity and constituents of municipal solid waste that contains lead contents such as electronic waste, lead batteries, lead-based paints, pipes, and plastics that are disposed in the dump site without segregation. In addition, it might be due to the age of the dumpsites.

The values of cadmium in 10 meters, 30 meters, and 60 meters away from the dump site were higher than the limit prescribed by Ethiopian environmental protection agency standard (0.5 mg/kg). In addition, cadmium values were higher than in the US EPA standard (1.4 mg/kg) at 10 meters and 30 meters away from the dump site. The finding was supported by other studies conducted in Adama and Maradi in Niger solid waste dump sites that revealed a higher average content of cadmium nearest to the dump site [5, 34]. This indicated that solid waste open dump site contributes to increasing the concentration of heavy metals in the nearest soil. Contrary to this, the finding in this study was lower than another finding in the Addis Ababa dump site [4]. The reason might be due to the difference in the age of dump sites and cadmium-containing wastes such as paints, batteries, and plastics were indiscriminately disposed of in the dump site.

The value of copper in the study area was higher than another finding in Addis Ababa (Ethiopia) and Isfahan province (Iran) [4, 12]. Moreover, the values were higher than the limit prescribed by United State environmental protection agency standard of 200 mg/kg. Copper values, however, were lower than the limit value prescribed by Ethiopian environmental protection agency standard of 500 mg/kg.

Zinc in the study area was higher than the finding in Addis Ababa (Ethiopia) and Maradi city (Niger Republic) dump sites which were 131.8 mg/kg and 97.98 mg/kg, respectively [4, 34]. It might be due to discharges of smelter slag, wastes, and use of commercial products such as fertilizers and wood preservatives that contain zinc that was observed at the dump site.

Zinc demonstrated considerable high mean values in the sample site, indicating that the soil around the dumpsite was largely polluted with this toxic metal. The fact that the concentration of Zinc in the soil around the dumpsite exceeded both US EPA, Ethiopian Environmental Protection Agency, and EU and UK limit values that are set in the range of 150 and 300 mg/kg indicates that the area is threatened by heavy metal pollution that may adversely affect environmental and ecological integrity in the long run.

## 5. Conclusion and Recommendations

The pH of soil was above 8 and basic which indicates the influence of solid waste dumped in the area. Similarly, EC was lower in 60 meters and higher in 10 meters and 30 meters sample sites, and the organic matter content of the soil showed a decreasing trend towards the dump site. The heavy metals such as cadmium, zinc, lead, and copper in soils have been found to be higher than EEPA and USEPA standards. The leachate sample was found to have higher concentration of heavy metals content such as lead, cadmium, manganese, nickel, and copper, and the entrance of this leachate to the adjacent river water especially near to dump site and downstream magnifies the problem as compared with the upper stream of the river from the dump site. The parameters exceeding the permissible limits of EEPA and WHO standards included pH, TDS, turbidity, BOD_5_, COD, manganese, and nickel. Consequently, the water of the stream has been polluted physically and chemically through the indiscriminate disposal of solid waste and discharge of leachate.

It is highly recommended that the municipality in Tepi town should invest in waste management and environmental protection activities. The soil in the study area needs different phytoremediation technologies. Indiscriminate waste discharge should be prohibited. Waste reduction, recycling, and reuse must be promoted, while at the same time, there is a critical need for the construction of a sanitary landfill or at least a controlled tipping site to manage the generated waste.

## Figures and Tables

**Table 1:** The physiochemical properties of leachate sample at Tepi town solid dump site.

Parameters	Leachate sample	EEPA (2003) standard	WHO (2004) standard
Temperature (°C)	32.9 ± 0.29	5–30	NA
pH	8.5 ± 0.12	6–9	6.5–8.5
EC (*μ*S/cm)	391.3 ± 0.01	1000	1400
TDS (mg/l)	782.5 ± 0.15	NA	500
Turbidity (NTU)	798.4 ± 0.5	NA	25
Nitrate (mg/l)	1.88 ± 0.01	50	30
Sulfate (mg/l)	98 ± 0.09	200	200
Potassium (mg/l)	20.1 ± 0.29	NA	12
Fluoride (mg/l)	1.71 ± 0.01	1	1.5
BOD (mg/l)	620.2	<5	<5
COD (mg/l)	935.33	5	<5

**Table 2:** The physicochemical properties of river water samples at Tepi town solid dump site along different sample locations and guideline values.

Parameters	US	DS1	DS2	EEPA (2003) standard	WHO (2004) standard
Temperature (°C)	22 ± 0.1	27.5 ± 0.2	27 ± 0.5	5–30	NA
pH	7.6 ± 0.21	8.1 ± 0.12	8.0 ± 0.1	6–9	6.5–8.5
EC (*μ*S/cm)	238.2 ± 0.2	281.3 ± 0.01	247.8 ± 0.02	1000	1400
TDS (mg/l)	446.3 ± 0.2	557.9 ± 0.1	495.7 ± 0.1	NA	500
Turbidity (NTU)	61.6 ± 0.01	144.0 ± 0.3	135.3 ± 0.7	NA	25
Nitrate (mg/l)	0.8 ± 0.01	1.72 ± 0.01	1.48 ± 0.01	50	30
Sulfate (mg/l)	16 ± 0.1	26 ± 0.8	63 ± 0.5	200	200
Potassium (mg/l)	8.5 ± 0. 05	12.1 ± 0.17	9.8 ± 0.15	NA	12
Fluoride (mg/l)	0.40 ± 0.01	0.88 ± 0.01	0.8 ± 0.01	1	1.5
BOD (mg/l)	7.9	31	12	<5	<5
COD (mg/l)	10.51	61.33	18.4	5	<5

**Table 3:** Heavy metal result of river water samples at Tepi town solid waste dump site along different sample locations and guideline values.

Parameters	US	DS1	DS2	EEPA (2003) standard	WHO (2004) standard
Cadmium (mg/l)	Bdl	Bdl	Bdl	0.005	0.003
Copper (mg/l)	Bdl	0.02 ± 0.95	0.018 ± 1.04	0.05–1.1	2
Lead (mg/l)	Bdl	Bdl	Bdl	0.1	0.05
Zinc (mg/l)	0.211 ± 0.2	0.39 ± 0.18	0.34 ± 0.2	0.5	0.05
Nickel (mg/l)	Bdl	0.08 ± 0.1	0.06 ± 0.13	0.1	0.02
Manganese (mg/l)	0.18 ± 0.01	0.4 ± 0.1	0.22 ± 0.1	0.3	0.1

**Table 4:** Heavy metal result of the leachate sample at Tepi town solid dump site.

Parameters	Leachate	EEPA (2003) standard	WHO (2004) standard
Cadmium (mg/l)	0.3 ± 0.01	0.005	0.003
Copper (mg/l)	0.26 ± 1.084	0.05–1.1	2
Lead (mg/l)	0.08 ± 0.1	0.1	0.05
Zinc (mg/l)	0.54 ± 0.2	0.5	0.05
Nickel (mg/l)	0.4 ± 0.1	0.1	0.02
Manganese (mg/l)	0.66 ± 0.04	0.3	0.1

**Table 5:** Heavy metal result of soil samples at Tepi town solid waste dump site along different sample locations and guideline values.

Soil sample site	pH	EC (*μ*S/cm)	Lead (mg/kg)	Copper (mg/kg)	Cadmium (mg/kg)	Zinc (mg/kg)
10 m	8.7 ± 0.21	4920 ± 1.04	57.56 ± 0.26	286.11 ± 0.2	2.26 ± 0.02	859.41 ± 0.02
30 m	8.4 ± 0.1	3490 ± 0.66	52.21 ± 0.02	204.06 ± 0.06	1.6 ± 0.01	623.93 ± 0.29
60 m	8 ± 0.1	1800 ± 0.5	3.26 ± 0.25	337.11 ± 0.01	0.53 ± 0.01	826.45 ± 0.01
US/EPA standard	6.5–8.5	1400	50–100	150–200	1.4	300
EEPA (2003) standard	6.5–9	1000	40	500	0.5	500

## Data Availability

The data used to support the findings of this study are included within the article.
